# Lightweight Road Adaptive Path Tracking Based on Soft Actor–Critic RL Method

**DOI:** 10.3390/s25196079

**Published:** 2025-10-02

**Authors:** Yubo Weng, Jinhong Sun

**Affiliations:** 1 Beijing-Dublin International College Electronic Information Engineering, Beijing University of Technology, Beijing 100124, China; w1140938954@163.com; 2 Department of Electrical and Electronic Engineering, The Hong Kong Polytechnic University, Kowloon 999077, Hong Kong

**Keywords:** speed-adaptive, road surface adaptive, soft actor-critic, path tracking, road surface detection, Stanley method

## Abstract

We propose a speed-adaptive robot accurate path-tracking framework based on the soft actor–critic (SAC) and Stanley methods (STANLY_ASAC). First, the Lidar–Inertial Odometry Simultaneous Localization and Mapping (LIO-SLAM) method is used to map the environment and the LIO-localization framework is adopted to achieve real-time positioning and output the robot pose at 100 Hz. Next, the Rapidly exploring Random Tree (RRT) algorithm is employed for global path planning. On this basis, we integrate an improved A* algorithm for local obstacle avoidance and apply a gradient descent smoothing algorithm to generate a reference path that satisfies the robot’s kinematic constraints. Secondly, a network classification model based on U-Net is used to classify common road surfaces and generate classification results that significantly compensate for tracking accuracy errors caused by incorrect road surface coefficients. Next, we leverage the powerful learning capability of adaptive SAC (ASAC) to adaptively adjust the vehicle’s acceleration and lateral deviation gain according to the road and vehicle states. Vehicle acceleration is used to generate the real-time tracking speed, and the lateral deviation gain is used to calculate the front wheel angle via the Stanley tracking algorithm. Finally, we deploy the algorithm on a mobile robot and test its path-tracking performance in different scenarios. The results show that the proposed path-tracking algorithm can accurately follow the generated path.

## 1. Introduction

Mobile robots are equipped with a variety of sensors, which not only provide rich environmental information but also offer high-precision pose estimation. By using three-dimensional (3D) mapping based on the LIO architecture, the vehicle can better understand its driving environment [1,2]. To achieve real-time six-degree-of-freedom pose estimation, a lightweight lidar odometry method is applied [3]. It has high accuracy and computational efficiency, and can reduce pose estimation errors through the SLAM framework; however, it does not consider point cloud distortion during motion. Reference [4] proposed a tightly coupled iterative Kalman filter algorithm for fusing IMU and LiDAR data in scenarios where the robot moves rapidly and encounters sparse features. However, this algorithm was limited to small-scale environments and did not incorporate loop detection, which may have led to cumulative errors in large-scale scenarios, thus affecting practicality. Y. Wang et al. proposed a prediction module to separately estimate and optimize the rotation and displacement directions, providing more accurate initial values for the back-end module [5]. However, the optimization process significantly increases computation time and complexity, and does not consider the effect of height information. Effective 3D mapping can provide essential environmental information for autonomous driving systems, and path planning can leverage this information for effective navigation decisions.

Path planning is a key component in the autonomous navigation of robots. Considering safety, driving efficiency, passenger comfort, and energy consumption simultaneously complicates the planning process [6,7]. Based on an improved RRT algorithm, the work in [8] proposed a strategy that added the endpoint cost to each node in the RRT* search, thereby reducing the path length of the random search and improving path smoothness. However, this study mainly focused on static environments and did not consider the shapes of surrounding obstacles. Reference [9] proposed a path-planning method based on RRT*, aiming to improve robots’ mission success rate under uncertain terrain conditions. Although the proxy model reduces computational cost, the introduced reliability constraints may increase overall computational complexity and affect real-time performance. Reference [10] used an artificial potential field (APF) method to improve the expansion point efficiency of the A* algorithm, successfully reducing the number of search nodes and improving obstacle avoidance efficiency. However, it did not smooth the path, potentially causing curvature discontinuities and affect the robot’s tracking efficiency. After path planning is completed, the robot needs to analyze the road surface information along the selected path to enhance environmental adaptability.

In actual operation, the friction coefficients of different road types affect the robot’s motion performance; thus, enhancing environmental perception capabilities has become urgent [11]. In reference [12], a multiscale convolutional attention network was introduced for the road detection task, which can more effectively capture information from occluded areas. However, this method mainly focused on road extraction in satellite images and failed to effectively segment road surface materials. An improved PointNet++ model capable of segmenting road irregularities and quantifying road roughness was proposed in [13], thereby improving the vehicle’s perception of surface unevenness and enhancing driving safety. However, this work mainly focused on road surface unevenness, did not consider road surface materials, and was not validated through real vehicle testing. A nonholonomic robot model with integrated vision was proposed in [14]; this model utilizes visual information to control vehicle motion and offers advantages such as low computational cost, simplicity, robustness, and finite-time convergence. A large-scale visual model can detect and identify obstacles on the road in real time, such as potholes, standing water, and stones, helping vehicles avoid potential risks during driving. In addition, the visual model can identify traffic signs and road markings, providing more comprehensive environmental information and assisting vehicles in decision-making.

As one of the core technologies for autonomous navigation of mobile robots [15], path tracking will continue to promote the application of mobile robots in more complex and diverse environments. Reference [16] adopted a reaction-based method to generate a path and used the pure-pursuit (PP) algorithm to achieve accurate path tracking. However, this study involved iterative optimization, which increased computational time and affected the real-time performance of the robot. In addition, the influence of road conditions and disturbances was not considered in the path-tracking process. Y. Tian et al. used important state information as input for reinforcement learning (RL) during the training process [17], which greatly improved the convergence efficiency of the training framework. They also designed a reward function to ensure tracking accuracy during autonomous driving and to improve driving safety. However, this study did not consider the impact of road surface materials and did not conduct real vehicle tests. In [18], a switching strategy for robot path tracking was adopted. In a known environment, a deterministic PP method was used; in an unknown and obstacle-filled environment, a deep RL-based method was employed for path tracking to better adapt to different environments. However, this scheme required the design of corresponding rules, and there was significant uncertainty in environmental assessment. Reference [19] used an RL path-tracking framework, which can effectively handle the effects of uncertainty and disturbance. Additionally, a new experience pool priority mechanism was designed to improve the reward mechanism, and a dynamic reward function was introduced to reduce computing resource consumption, thereby accelerating convergence and avoiding local minima. However, this study did not incorporate robot body information, focused only on path tracking, and did not consider factors such as efficiency and energy consumption in the tracking process.

This paper proposes a velocity-adaptive robot path-tracking framework based on the SAC algorithm, aiming to achieve efficient and accurate path tracking and thus promote the application and development of mobile robots in complex environments. The framework integrates a variety of advanced technologies. In particular, the road state detection results from the large-scale visual model are fed into the reinforcement learning (RL) algorithm as feature values, significantly enhancing the perception and decision-making capabilities of the autonomous driving system. Specifically, the visual model processes real-time images to extract the road state, obstacle information, and other relevant features. These extracted features are then converted into state representations for the reinforcement learning model, forming the state space. Based on the current state information, the reinforcement learning algorithm optimizes the vehicle’s control strategy—including acceleration, deceleration, and steering—to adapt to different road conditions. During driving, the vehicle continuously collects new visual and feedback data, updating and optimizing the control strategy through reinforcement learning, which improves the adaptability and robustness of the system. The main contributions of this work are as follows:

- The 3D environment mapping and localization method based on LIO is used to achieve stable robot pose output. In addition, by combining a gradient descent-based obstacle avoidance and path smoothing algorithm, the computational cost is reduced and planning efficiency is improved, all while satisfying the robot’s kinematic constraints.
- A U-Net-based classification model is employed to perform detailed classification of road scenes, thereby enhancing the robot’s ability to perceive the road surface. This allows the robot to slow down in advance in complex environments and improves safety.
- Leveraging the powerful learning capability of the ASAC algorithm, the proposed approach better represents the vehicle’s dynamic model and incorporates road surface information to enable road surface perception. This allows the robot to slow down in advance on slippery roads and increase its driving speed on dry and normal roads, thereby ensuring safety and improving movement efficiency.
- Our STANLY_ASAC controller requires only the nominal values of vehicle parameters, rather than their exact values, to generate optimal acceleration and convert it into speed commands. In addition, the optimal look-ahead distance is adaptively determined by the geometry-based Stanley controller to obtain the optimal front wheel angle, which greatly reduces computational load and improves tracking efficiency.

## 2. Problem Formulation

**Figure 1:**
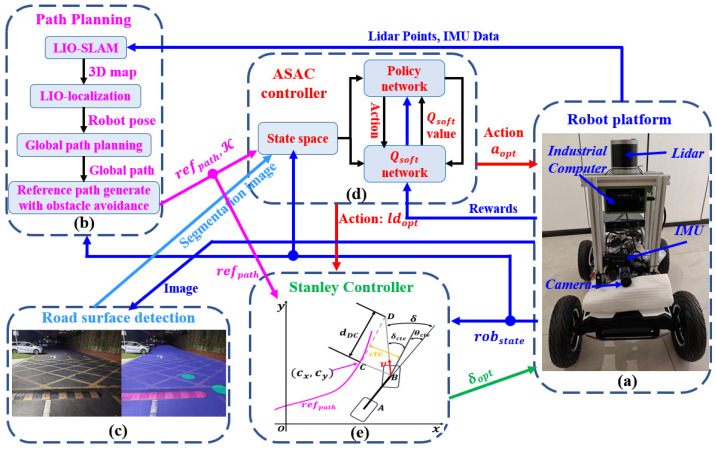
Structure of the whole system. (**a**) Robot plantform; (**b**) Path Planning; (**c**) Road surface detection; (**d**) ASAC controller; (**e**) Stanley Controller.

A lightweight, road-adaptive path-tracking framework based on ASAC and Stanley methods is shown in Figure 1. The robot state *rob*_state_ includes the coordinates of the vehicle center (x,y), speed *v*, acceleration *a*, and heading angle θ. The main hardware components used in this work and their specifications are shown in Table 1.

1. The Robosense 32-line LiDAR is used to output point cloud information. The IMU-GPS integrated inertial navigation system provides pose information. The Hikvision MV-CA013-21UC color camera (Hikvision, Hangzhou, China), equipped with a 6-megapixel industrial lens, is mainly used to capture images of the surrounding environment.
2. For the path planning module, a 3D map of the environment is first constructed based on the LIO architecture, which is also used to obtain the pose of the mobile robot at an output frequency of 100 Hz. After stable pose information is obtained, a global path-planning method based on the RRT algorithm is used to directly generate a point-to-point global path according to the current task. Subsequently, a local path that can avoid obstacles and satisfy the robot’s kinematic constraints is generated using an improved A* algorithm combined with the gradient descent method, guiding the robot to reach the target point safely and efficiently. Finally, the generated reference path, *ref*_path_, and the curvature κ of each path point are input to the ASAC controller.
3. We use a vision-based road classification algorithm to semantically segment different road surfaces and obtain information about their corresponding friction coefficients (*Road*_seg_map_). This information is then input into the ASAC controller, enabling it to perceive road conditions in real time and adjust the speed accordingly.
4. In the ASAC controller, we leverage its powerful network learning capability to adaptively adjust the acceleration and look-ahead distance in real time, based on input information such as the reference obstacle avoidance path, road classification results, and vehicle status.
5. The Stanley tracking algorithm is used for path tracking control. Based on *rob*_state_ and *ref*_path_, the required front wheel steering angle δ is calculated and dynamically adjusted in real time according to vehicle speed and path curvature. This scheme features simple and easy-to-implement control logic, and exhibits strong robustness to external disturbances and environmental changes. It can also effectively adjust the vehicle steering angle under complex road conditions.

## 3. Three-Dimensional Mapping and Path Generation

### 3.1. Three-Dimensional Environment Modeling and Positioning Based on LIO

**Figure 2:**
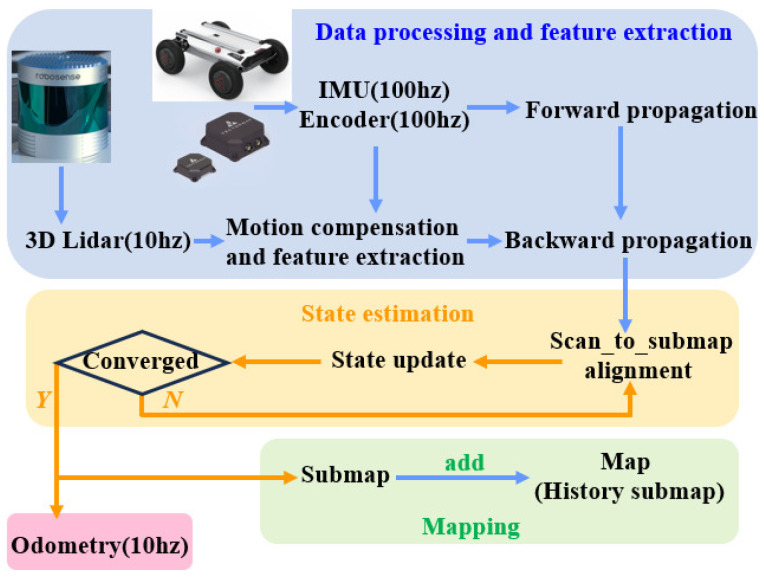
Control flow of the 3D mapping.

As there is a time difference between the starting point and the endpoint when the LiDAR completes one full rotation, the generated point cloud may contain errors. Therefore, as shown in Figure 2, we fuse data from the IMU and the robot’s odometer to estimate the robot’s motion state [12]. We then use a backward alignment method to align the initial point cloud with the final point cloud after motion integration, thereby compensating for motion distortion in the laser point cloud. Subsequently, features are extracted, and forward integration using the IMU is performed to obtain the initial position of the point cloud. Next, the iterative closest point (ICP) algorithm is used to align the point cloud with the sub-map and to obtain the rotation and translation of the current frame. Finally, the updated sub-map is integrated into the global map, thereby achieving 3D mapping of the surrounding environment.

To meet the requirements of robot control, the output frequency of the odometer needs to be increased to 100 Hz. To this end, we use the IMU pre-integration method to integrate the motion between LiDAR frames, which can more effectively handle noise and deviations in the IMU and reduce cumulative errors. In addition, when the matching between the LiDAR and the submap converges, the model between adjacent frames can be further optimized. After each LiDAR frame matching and optimization is completed, and before the next LiDAR frame arrives, the most recently updated optimization parameters can be used to estimate the robot’s state. This not only provides a more accurate initial value for the next LiDAR frame but also improves the convergence speed of the ICP algorithm, thereby achieving accurate and stable robot pose output that meets the requirements of the path tracking module.

### 3.2. Global Path Generation

For point-to-point task planning, we first rasterize the 3D map into a 2D grid map. Then, we use the RRT algorithm [20] for global path planning, which is particularly suitable for spatial path planning problems. RRT rapidly explores the space via random sampling to find a feasible path from the start to the goal. The process mainly includes the following steps:

1. Initialization: Initialize the tree with the start node located at the starting position.
2. Random sampling: Generate a random sample point in the search space.
3. Nearest neighbor search: Find the node in the tree that is closest to the sample point.
4. Expand the tree: Expand the tree by creating a new node from the nearest node in the direction of the sample point. The expansion step size is usually fixed to ensure gradual tree growth.
5. Collision detection: Check whether the new node collides with any obstacles in the environment. If there is no collision, add the new node to the tree.
6. Check the target: If the new node is sufficiently close to the target position, a path has been found.
7. Repeat steps 2 to 6 until a path is found or the maximum number of iterations is reached.

### 3.3. Path Smoothing

The path corresponding points $\vec{s_{z}}={\left[x_{z},y_{z}\right]}^{T}$, where z=1,2,…,N, are generated based on the improved A* algorithm mentioned in our previous work in [21]. To further optimize the path curvature and enhance driving safety and comfort, a path smoothing method based on the conjugate gradient descent algorithm is used. By minimizing the path curvature cost function *J*_cur_, as defined in (1), which calculates the change in angle between adjacent vertices for 2≤z≤N−1, the resulting path becomes smoother. This reduces unnecessary steering and improves the overall driving experience.

(1) $$J_{cur}=\sum_{z=1}^{N-1}{\left(\left(S_{z}-S_{z-1}\right)-\left(S_{z+1}-S_{z}\right)\right)}^{2}$$

Starting from an initial path, the gradient at each point is calculated, and the search is performed along the negative gradient direction with a convergence condition set. When the convergence condition is met, the search stops; otherwise, the iteration continues. In this way, our path optimizer gradually improves the path and ultimately obtains a smooth path that satisfies the vehicle’s kinematic constraints. The specific steps are as follows:

1. Initialize the waypoints to the original waypoints.
2. For each path point $S_{z}$, calculate the gradient of the objective function:
  (2) $$S_{z}\nabla J_{cur}=2\left(S_{z-1}-2S_{z}+S_{z+1}\right)$$
3. Waypoint Updates:
  (3) $$S_{z}=S_{z}-\lambda \nabla J_{cur}\left(S_{z}\right)$$
4. Repeat the above steps until the objective function converges or the maximum number of iterations is reached.
  where λ is the learning rate, which controls the step size of the update. Choosing an appropriate learning rate λ is critical: a large value may cause instability; if it is too small, it will slow down convergence. In this paper, the learning rate λ is set to 0.001.

## 4. Pavement Classification Algorithm

Different road types typically correspond to different friction coefficients. In this work, we do not directly estimate the friction coefficient of the road surface. Instead, we classify road surface types and incorporate this classification information into the ASAC controller. This enables the controller to adaptively adjust to varying road conditions, thereby improving path tracking accuracy. The U-Net network, which has a symmetrical encoder–decoder architecture [22], is used to classify the actual road type in real time. The friction coefficient is then dynamically mapped based on the identified road type and input to the controller, enabling the control strategy to adapt to real-time changes in friction. Semantic segmentation is performed using a dataset containing 701 frames from RTK. We divide the road surface materials into 13 different categories: black—everything not related to the road; light blue—roads with asphalt surfaces; greenish blue—various pavements; peach/light orange—unpaved roads; white—road markings; pink—speed bumps; yellow—cat’s eyes; purple—storm drains; cyan—manhole covers; dark blue—patches on asphalt roads; dark red—water puddles; red—potholes; and orange—cracks.

**Figure 3:**
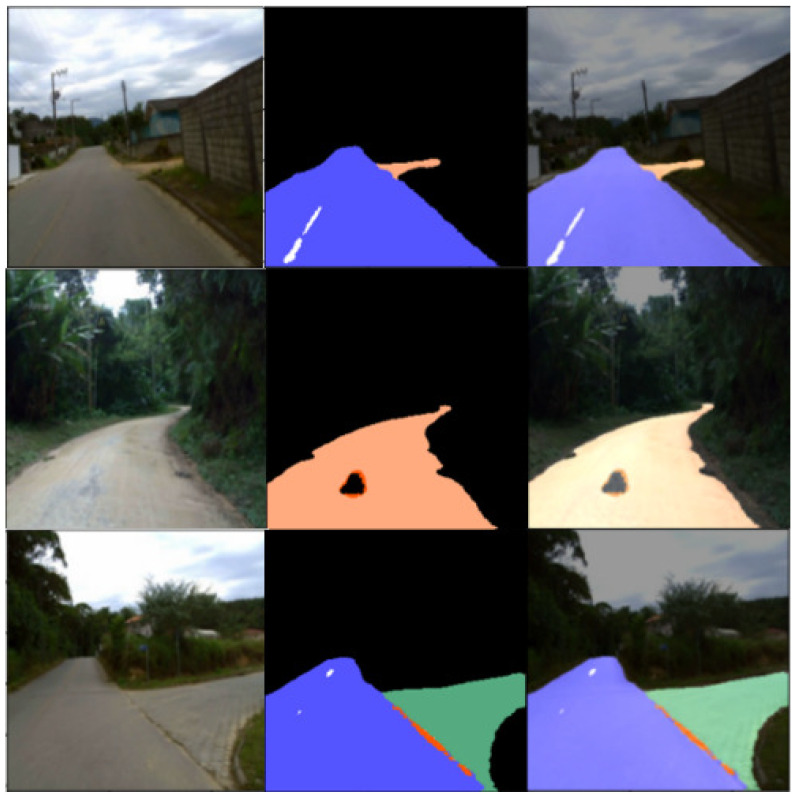
Results of the RTK image processing.

For testing, we separated most of the frames by category and we present the results in Figure 3. The original image is shown on the left, and the prediction results are in the middle, where different road types are represented as color images generated by the network, each corresponding to a specific road category. The rightmost part displays the detection result after the prediction output is mapped onto the original image with the background removed. The total number of parameters in the network is 1,941,309. The input image size is 256×256×3, and the output is 256×256×13, indicating the category to which each pixel belongs. Table 2 shows the specifications of the U-Net, including each layer and its corresponding input and output parameter dimensions.

To improve data diversity and model robustness, we employed various data augmentation strategies during our experiments, including horizontal and vertical flipping, rotation within $\pm 15^{\circ }$, scaling from 0.8 to 1.2, color jittering (brightness, contrast, and saturation), and random cropping. These augmentation methods effectively expanded the distribution of the training samples and provided strong data support for subsequent performance improvements. Details are given in Table 3, where precision is defined as the ratio of correctly predicted positive pixels to all predicted positive pixels, and recall represents the proportion of true positive pixels that are correctly identified.

## 5. ASAC Controller and Path Tracking

### 5.1. ASAC Controller

The ASAC controller effectively handles interference and accurately reflects vehicle dynamics, thereby generating reasonable speed control gains and improving tracking stability. It is able to adapt to changes in vehicle dynamics parameters and can improve tolerance to model uncertainties by adjusting control gains online.

**State:** $\mathrm{rob}state\left(x,y,\theta ,v_{x},v_{y}\right)$, *Road*_seg_map_, the lateral tracking error cte and the heading tracking error eψ, $\left({ref}_{x0},{ref}_{y0},K_{0},{ref}_{x1},{ref}_{y1},K_{1},\ldots ,{ref}_{x7},{ref}_{y7},K_{7}\right)$.

**Action:** The output action variables of the ASAC algorithm are $a\in \left[0,a_{max}\right]$, and the control gain *k*. The setting of *k* is crucial to vehicle response: if *k* is too large, the vehicle becomes overly sensitive to lateral errors and is prone to oscillation; if *k* is too small, the response is sluggish and it becomes difficult to correct deviations in a timely manner. A reasonable selection of *k* is key to ensuring both stability and accuracy in path tracking.

**Reward:** To more realistically reflect and optimize tracking, the reward function in this design focuses on both tracking performance and ride comfort. The maximum penalty is $-r_{max}$, which is set to 20 in this work and serves to eliminate violations of the system requirement definition.

1. During the control process, the controller should minimize both lateral and heading errors, with particular emphasis on lateral errors. To help the controller determine whether the vehicle has sufficient space to turn safely, half of the vehicle body width,$w={car}_{width}/2$, serves as an important reference for evaluating tracking performance.
  (4) $$r_{cte}=\left\{\begin{array}{l} k_{1}*\left(w-cte\right)\;\;\;\;cte\;<\;\;w\\ -r_{max}\;\;\;\;\;\;\;\;\;\;\;\;cte\;\geq \;\;w \end{array}\right.$$
  (5) $$r_{e\psi }=\left\{\begin{array}{l} e^{-k_{2}\left|e\psi \right|\;\;\;\;\;\left|e\psi \right|\;<\;\frac{\pi }{2}}\\ -r_{max}\;\;\;\;\;\left|e\psi \right|\;\geq \;\frac{\pi }{2} \end{array}\right.$$
2. The robot moves within the road’s speed limit. A higher speed can reduce the time required to travel the same route, thereby improving tracking efficiency, which is primarily reflected in speed. At the same time, to enhance ride comfort, frequent and large accelerations or decelerations should be avoided as much as possible.
  (6) $$r_{v}=\left\{\begin{array}{l} k_{3}*\left(v-v_{min}\right)\;\;\;\;\;\;\;v\;>\;v_{min}\;\\ -r_{max}\;\;\;\;\;\;\;\;\;\;\;\;\;\;\;\;v\;\leq \;v_{min} \end{array}\right.$$
  (7) $$r_{a}=\left\{\begin{array}{l} k_{4}*\left(a_{max}-a\right)\;\;\;\;\;\;\;a<\;a_{max}\;\\ -r_{max}\;\;\;\;\;\;\;\;\;\;\;\;\;\;\;\;a\;\geq \;a_{max} \end{array}\right.$$

Finally, the total reward is given as (8).

(8) $$R=r_{cte}+r_{e\psi }+r_{v}+r_{a}$$

This study systematically designed the hyperparameter tuning process for the SAC algorithm to ensure reproducible results. We prioritized key hyperparameters such as the learning rate, discount factor, and target network update frequency, and set them appropriately. See Table 4 for detailed parameter configurations.

### 5.2. Network of the ASAC

**Figure 4:**
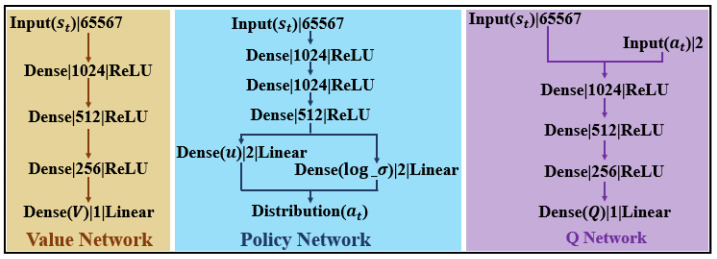
Detailed network architecture showing input types, output dimensions, and activation functions for each layer.

Figure 4 shows the network structure, including the input types, output dimensions, and the corresponding activation functions of each layer. “Linear” indicates a direct linear output without an activation function, while “Dense” refers to a fully connected layer. We use the Q value as the critic and update the network parameters using temporal difference learning. The update equations for the critic and actor are given by (9) and (10), respectively.

(9) $$\begin{array}{c} Q\left(s,A\right)\leftarrow Q\left(s,A\right)-\lambda_{Q}\left(Q\left(s,A\right)-\left(R+\tau M\right)-\beta N\right)\\ M={min}_{i=1,2}Q\left(s^{\prime }A^{\prime }\right),N=\log\pi \left(A^{\prime }|s^{\prime }\right)\; \end{array}$$

(10) $$\pi \left(A|s\right)\leftarrow \pi \left(A|s\right)-\lambda_{\pi }\left(\nabla_{\pi }\left(\log\pi \left(A|s\right)Q\left(s,A\right)-\beta \log\pi \left(A|s\right)\right)\right)$$

where $Q\left(s,A\right)$ denotes the value of taking action *A* in state *s* as output by the critic network, τ is the discount factor, $\lambda_{Q}$ and $\lambda_{\pi }$ are the learning rate of the critic and actor networks, respectively; β is the coefficient of the entropy regularization term; M is computed by two independent critic networks and represents the minimum value of the action to be taken in the next state; N represents the probability density of taking an action in the next state; $\pi \left(A|s\right)$ is the output of the actor network; and $\nabla_{\pi }$ is the gradient of the actor network.

### 5.3. Path-Tracking Controller

The Stanley-based tracking controller shown in Figure 1 sets the reference point at the center of the front wheel to achieve global convergence in path tracking, and the error decay rate is not affected by the vehicle speed. It ensures heading correction and position error correction, and the generated steering angle remains within the limits of the robot’s dynamics. It acts as a safety net to ensure that the control output is physically feasible and to enhance the system’s robustness to dynamic differences. These considerations ensure the effectiveness and safety of the controller during path tracking.

**Figure 5:**
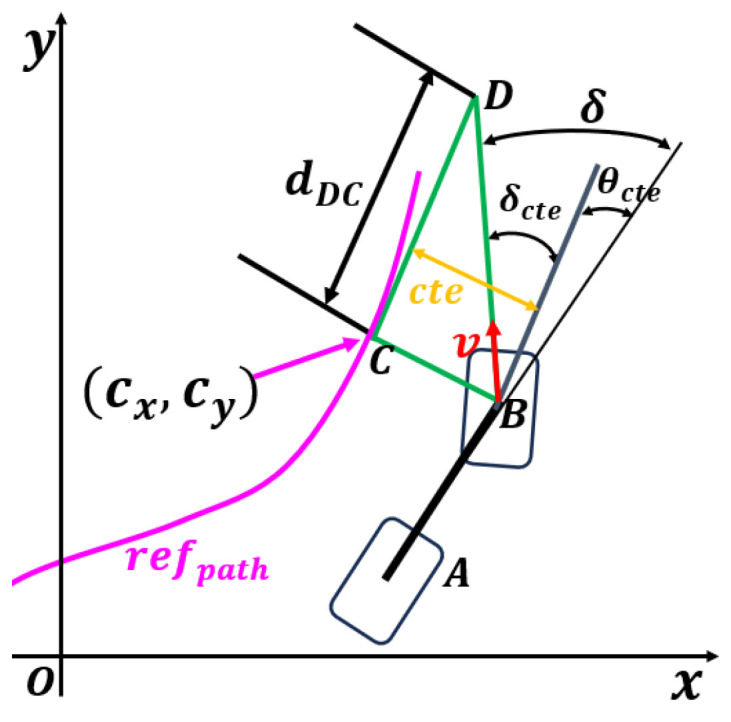
Geometric construction of triangle BCD for derivation of the lateral error angle and distance in path tracking.

To eliminate the heading error relative to the path, set $\delta \left(t\right)=\theta_{cte}\left(t\right)$. Assume that the lateral error of the robot is zero, i.e., cte=0. To achieve steering and eliminate the lateral error cte, triangle BCD can be constructed as shown in Figure 5. Take the nearest point on the tracking path to the front wheel center as *C* and determine point *D* at the intersection along the direction of the velocity. In this way, the angle $∠BDC=\delta_{cte}$ generated by cte can be obtained. Define the distance of DC as $d_{DC}$, then Equation (11) can be derived.

(11) $$\tan\left(\delta_{cte}\right)=\frac{cte}{d_{DC}}$$

As $d_{DC}$ is a speed-related quantity, introduce the ratio $\xi =v/d_{DC}$. Thus, the front wheel angle can be expressed as in Equation (12). For ξ, the action is generated by reinforcement learning. The resulting steering angle is always within the vehicle dynamics range and is constrained to $\delta \left(t\right)\epsilon \left[\delta_{min},\delta_{max}\right]$.

(12) $$\delta \left(t\right)={tan}^{-1}\left(\frac{\xi *cte(t)}{v(t)}\right)$$

## 6. Results and Discussion

Five training maps, ranging from simple to complex, were designed using the splicing function of CarMaker. The first map is a straight line to help the controller learn basic acceleration and deceleration operations. The second map contains large-radius curves, focusing on training the controller’s turning ability. The third map consists mainly of sharp turns, emphasizing the learning of sharp cornering maneuvers. The fourth and fifth maps combine various turning radii and straight sections to improve the controller’s adaptability to different map layouts. In addition, the road friction coefficient is randomly reduced in certain areas to enhance the controller’s robustness to varying road conditions. We also introduce a redundant controller, KMPC, to ensure safety and to enhance data collection and training for scenarios with large lateral errors. Once the gap between simulation and reality is sufficiently narrowed, the redundant controller will be removed to improve system efficiency.

### 6.1. 3D Mapping

**Figure 6:**
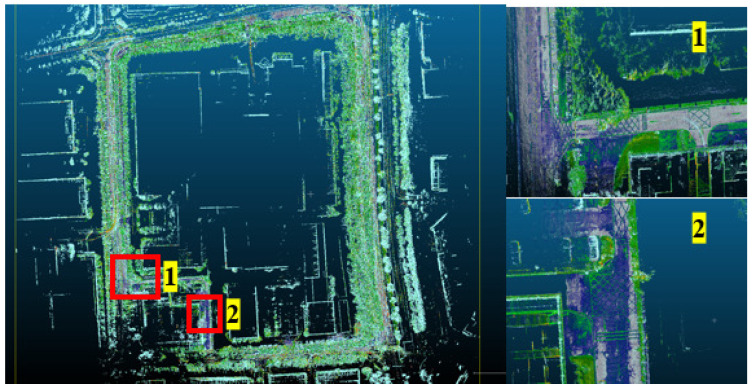
Overview of the 3D map for a 480 m × 353 m outdoor area.

A 3D map of the outdoor environment is shown in Figure 6, covering an area of 169,440 square meters, with a length of 480 m and a width of 353 m. In addition, two locations on the generated map are zoomed in, and the enlarged images clearly show various traffic elements of the environment, such as no-parking areas and directional guidance.

**Figure 7:**
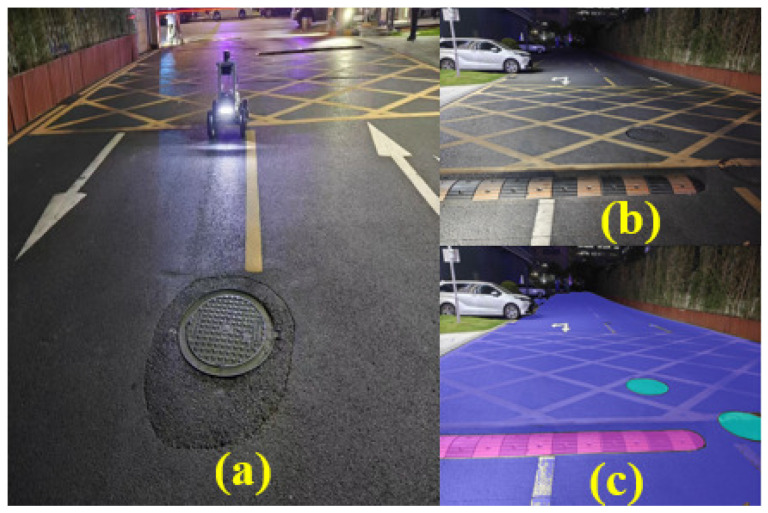
(**a**) Sensor platform, (**b**) real-time camera images, and (**c**) U-Net-based ground material segmentation results.

Figure 7a shows the main robot, which is equipped with sensors. Figure 7b presents the real-time image captured by the camera at a resolution of 640×480. Figure 7c displays the result of U-Net segmentation, where blue represents normal ground, pink indicates speed bumps, and light green denotes manhole covers. The segmentation of different ground materials is effective, and the results will be provided to the subsequent path-tracking module to enable accurate road surface information acquisition.

### 6.2. Path-Tracking Performance

**Figure 8:**
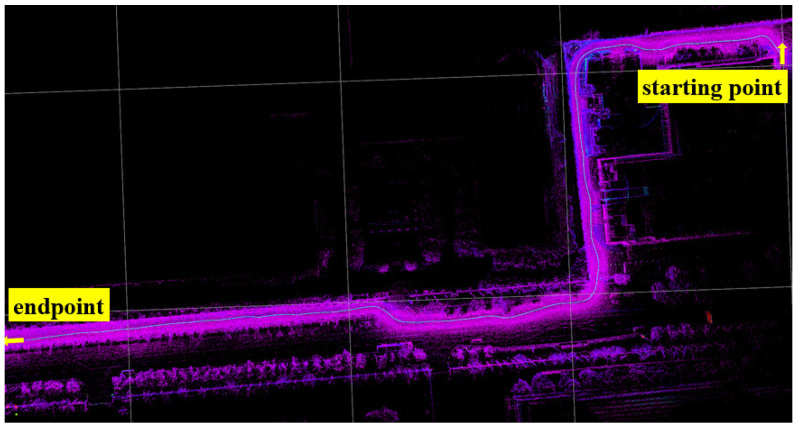
Selected path.

**Figure 9:**
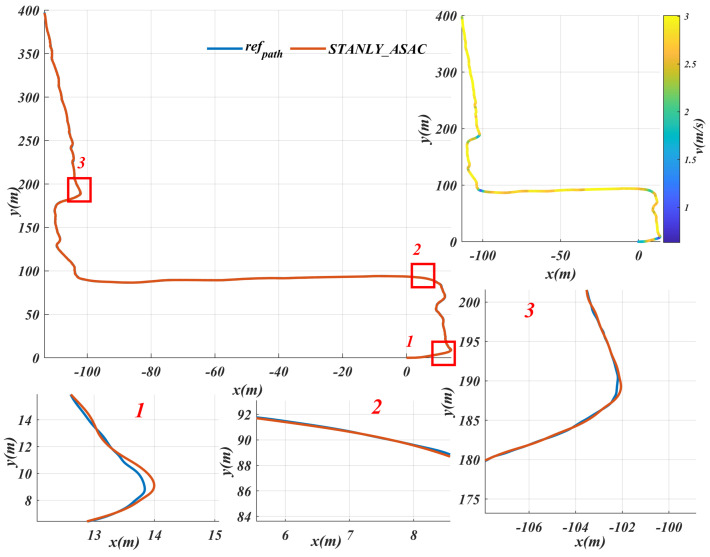
STANLY_ASAC path tracking results based on the reference path and speed change details in the key turning area.

Figure 8 shows a planned path selected from the established map, and the path-tracking results of the proposed solution are also illustrated in Figure 8. Due to the limitations of robot dynamics, the maximum speed is set to $3\;m/s\;\left(10.8\;\mathrm{km}/h\right)$. Figure 9 shows the STANLY_ASAC path tracking results based on ${ref}_{path}$, with three locations magnified. It can be observed that when the robot enters a turning area, it slows down in advance to ensure tracking accuracy and accelerates before completing the turn to improve driving efficiency. Throughout the entire driving process, the vehicle’s speed is maintained at the maximum speed of 3 m/s most of the time, which not only ensures tracking accuracy but also allows the robot to reach the target point smoothly at maximum speed.

**Figure 10:**
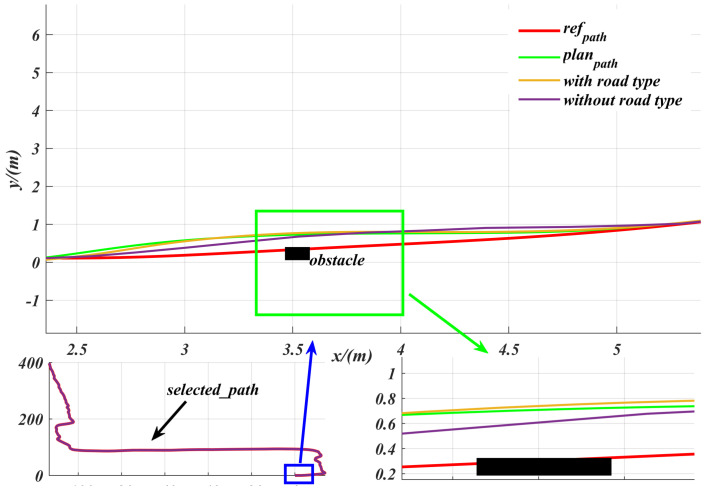
Comparison of path tracking performance using Hybrid A* path planning and different control algorithms in an unpaved road obstacle environment: zooming in on the blue box area gives a more clearer view of the control performance with obstacles.

Obstacle avoidance experiments are not only a key step in testing and optimizing the performance of path planning methods, but also an important guarantee for ensuring the safe and efficient operation of robots in complex, high-dimensional environments [23]. In our experiments, we used the Hybrid A* algorithm [15] to plan paths around obstacles encountered during driving. As shown in Figure 10, by setting the road type to “unpaved” in the marked area, we observed that Hybrid A* successfully generates a feasible path that avoids obstacles. The experimental results demonstrate that the proposed control algorithm is capable of accurately tracking the path planned by Hybrid A* in real time. Notably, on unpaved road sections, the controller’s lateral deviation is significantly reduced after incorporating road information, compared to the STANLY_ASAC algorithm, which does not utilize road information. This demonstrates higher tracking accuracy and stability. The final results show that the proposed control algorithm can effectively avoid obstacles regardless of whether road information is incorporated. However, incorporating road information further reduces tracking error and significantly improves the robustness and stability of the controller.

### 6.3. Control Quantity Generation During Path Tracking

**Figure 11:**
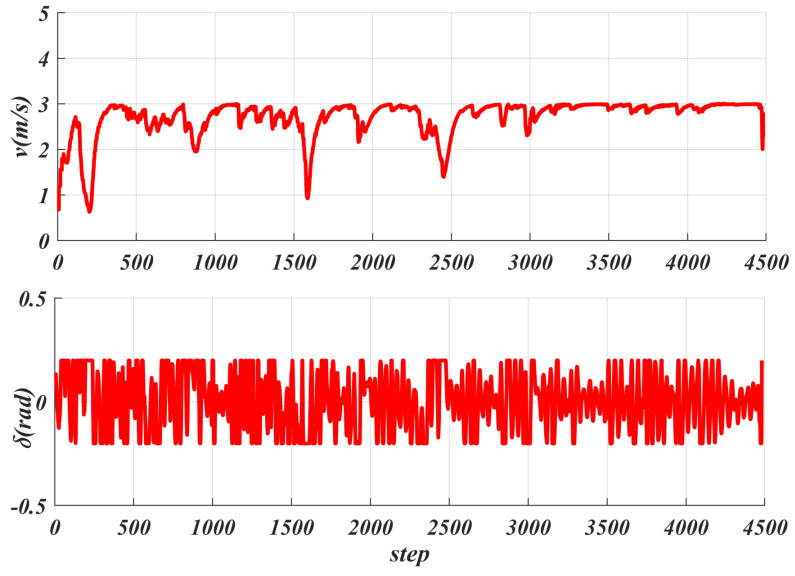
Real−time tracking speed and front wheel angle change curve under robot acceleration control.

**Figure 12:**
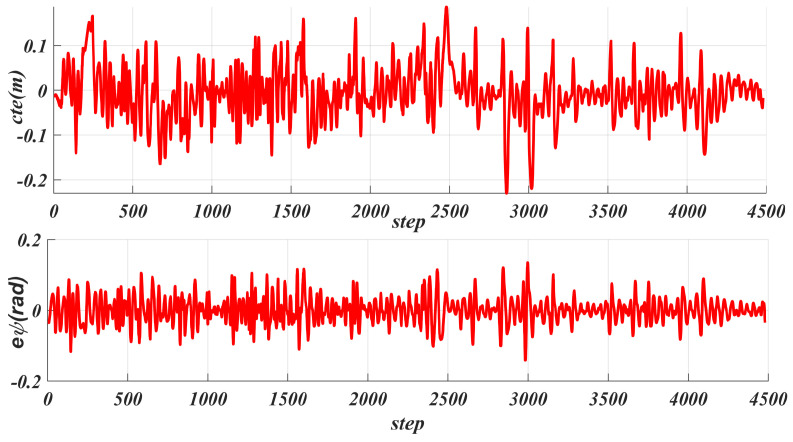
Statistical analysis of lateral error and heading error during robot path tracking.

Figure 11 shows the real-time tracking speed generated based on acceleration, with an average speed of 2.714 m/s and a maximum speed of 3 m/s, which is the maximum speed limit set for the robot. Each step represents one calculation cycle. Additionally, Figure 10 shows the front wheel angle control during tracking, with an average value of 0.087 rad (about $5^{\circ }$) and a maximum value of 0.2 rad (about $11^{\circ }$) set, where one step indicates one calculation cycle. Figure 12 shows the cte generated during the tracking process, with an average value of 0.0425 m and a maximum value of 0.23 m. For the heading deviation eψ given in Figure 12, its average value is 0.0275 rad and its maximum value is 0.1411 rad.

### 6.4. Disturbance in Path Tracking

**Figure 13:**
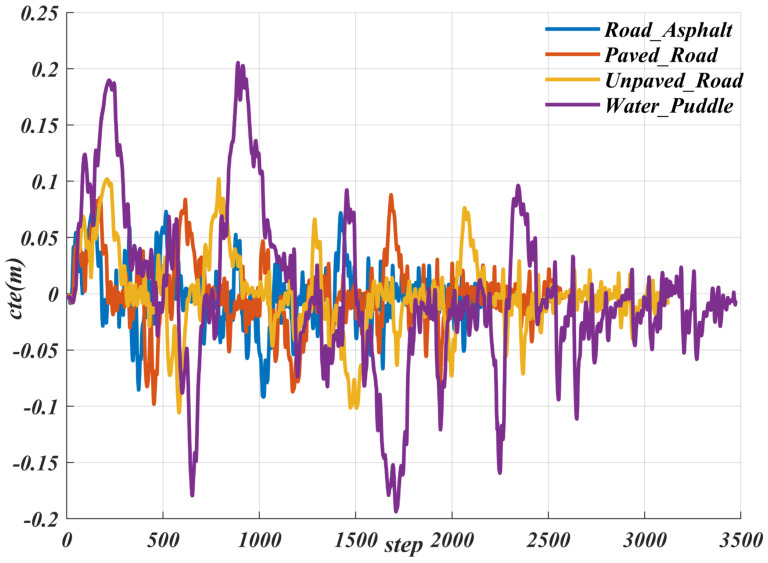
Result of the cte for various road conditions.

We imported the actual path into CarMaker and selected four common road types to verify the algorithm’s adaptability to different road surfaces. As shown in Figure 13 and Table 5, for the Road_Asphalt and Paved_Road road surfaces, our method performed similarly, with the maximum cte less than 0.1. For the Unpaved_Road road surface, the maximum cte did not exceed 0.11. Although on the Water_Puddle road surface, due to the significant reduction in friction coefficient, the maximum cte exceeded 0.2—nearly twice that of the previous three road surfaces—it was still within the safe range of path planning, thereby ensuring safety during driving. The proposed controller also demonstrates strong adaptability to external road disturbances. As shown in Figure 13, even under complex road conditions such as water puddles, the maximum cte does not exceed 0.25, consistently remaining within the safe distance set by the path planner. These results indicate that the system can effectively ensure driving safety under various road disturbances.

**Figure 14:**
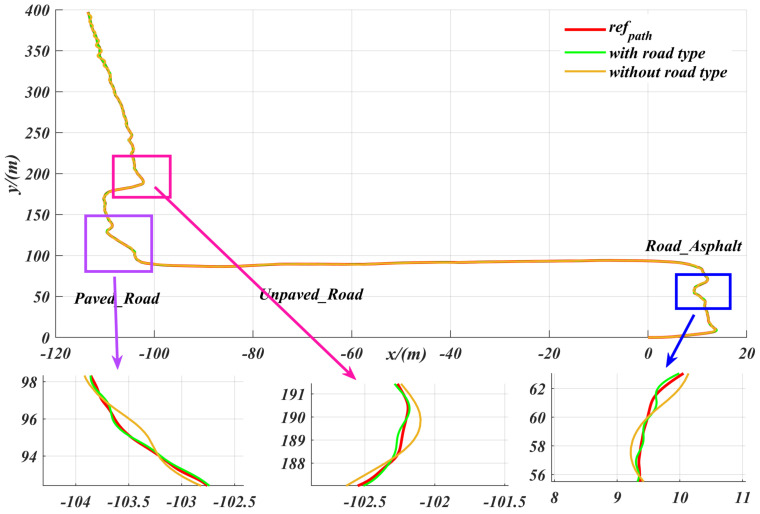
Comparison of the effects of road classification information on the lateral deviation in path tracking with the ASAC controller.

Different road conditions correspond to different friction coefficients, which in turn introduce external disturbances during the robot’s path tracking process. The algorithm proposed in this paper first classifies the road surfaces and then employs the ASAC controller for adaptive control. The path was imported into CarMaker, with different road surface types set at various locations. We then compared scenarios with and without road surface classification. As shown in the Figure 14, the tracking accuracy of the proposed controller, as measured by lateral deviation, was significantly reduced (i.e., lateral deviation was greater) in the scenario without road surface classification. This demonstrates that incorporating road surface classification information can significantly improve path tracking performance. This paper focuses on the role of road surface information in enhancing robot path tracking performance. Even in unknown scenarios where such information is unavailable, the proposed control algorithm maintains reliable path tracking, keeping the maximum cte within a safe range. For instance, on slippery surfaces such as water puddles, the algorithm’s maximum cte does not exceed 0.3 m, even in the presence of semantic segmentation errors. These results demonstrate the algorithm’s robustness to segmentation errors and its safety for practical applications. The main purpose of this study is to improve the accuracy of path tracking by introducing road surface information. However, the experimental results show that even in the absence of such information, the proposed controller can still achieve satisfactory path tracking performance, demonstrating strong adaptability and robustness.

### 6.5. Comparison with Other Control Methods

To better demonstrate the path-tracking performance of this solution, we selected several comparison algorithms for the same path: a model predictive control algorithm based on dynamic constraints (DMPC), a model predictive control algorithm based on kinematic constraints (KMPC), a quadratic linear regulator control algorithm based on dynamic constraints (DLQR), a pure-pursuit algorithm with a fixed look-ahead distance, and a PID control algorithm. Since these comparison algorithms are only used for path tracking, they primarily generate the front wheel angle and do not involve speed generation. Therefore, in this paper, the final speed generated by the STANLY_ASAC algorithm is used as the control speed for these algorithms during path tracking. The parameters of these controllers are given in Table 6.

**Figure 15:**
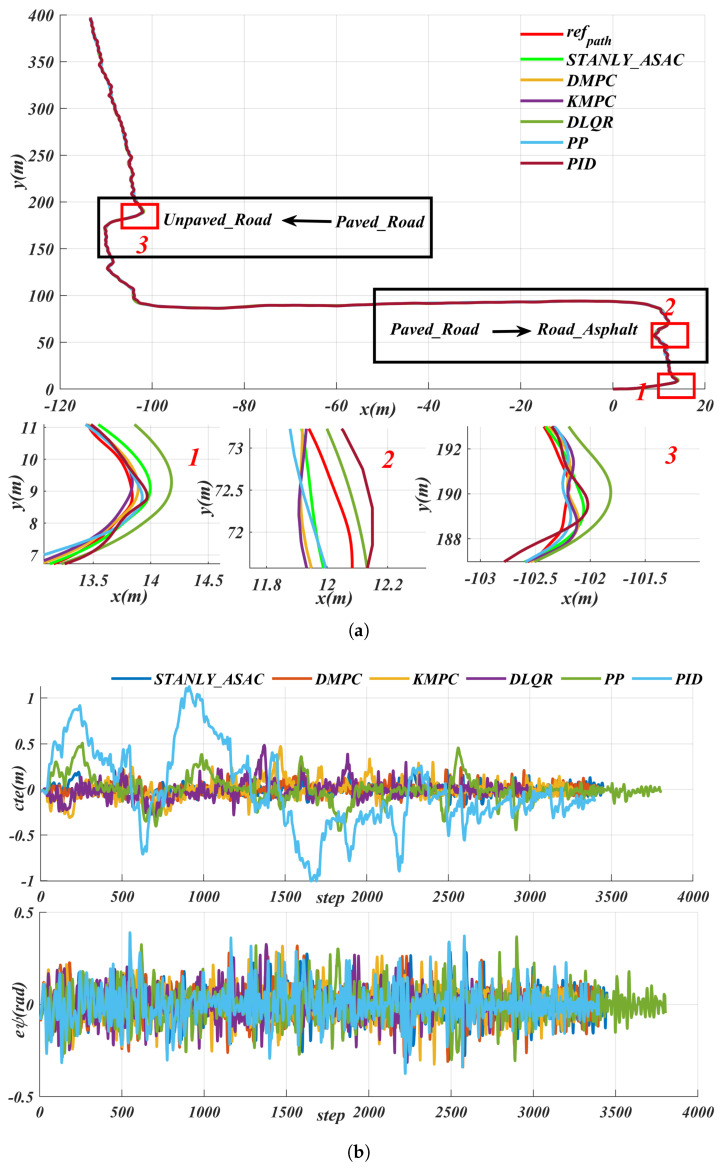
Comparative path−tracking performance of different control algorithms−lateral error (cte) and angular deviation under identical path and speed conditions: (**a**) path. (**b**) tracking error.

Figure 15 shows the path-tracking results of different control algorithms under the same path and speed conditions, including the lateral error (cte) and angular deviation performance for each algorithm. The experimental results demonstrate that the proposed STANLY_SAC reinforcement learning algorithm exhibits superior adaptability and robustness in complex road environments. Specifically, the maximum cte values of STANLY_SAC and dynamic-based model predictive control (DMPC) are 0.2352 and 0.2403, respectively, with average values of 0.0668 and 0.0771. Both the mean and maximum errors of STANLY_SAC are lower than those of DMPC. Furthermore, while the DMPC algorithm relies on precise robot dynamic parameters—which are often difficult to obtain in real-world environments—STANLY_SAC does not require accurate models, demonstrating its advantages in situations with parameter uncertainty or environmental fluctuations. For the kinematic-based model predictive control (KMPC) and discrete linear quadratic regulator (DLQR) algorithms, their maximum cte and average cte reached (0.4732, 0.4859) and (0.1341, 0.1554), respectively, which are almost double those of STANLY_SAC. This indicates that traditional model-based controllers are significantly less adaptable in complex road conditions. Due to its fixed look-ahead distance, the PP algorithm performs well only on straight sections; when faced with complex scenarios, its maximum and average cte rise to 0.4952 and 0.2385, respectively, indicating limited adaptability. The PID algorithm’s maximum cte reaches as high as 1.12, which severely impacts driving safety. In terms of angular deviation, both STANLY_SAC and DMPC are able to limit the maximum deviation to within 0.20 rad, whereas the other algorithms, due to their large lateral errors, generally exceed 0.30 rad. Particularly in complex scenarios involving extreme conditions such as sharp turns and U-turns, STANLY_SAC consistently keeps the lateral error (maximum cte) within 0.250 rad, effectively ensuring the safety and stability of path tracking. In summary, reinforcement learning algorithms demonstrate greater adaptability and robustness in the face of changing and complex road environments, outperforming traditional model-based control methods.

**Figure 16:**
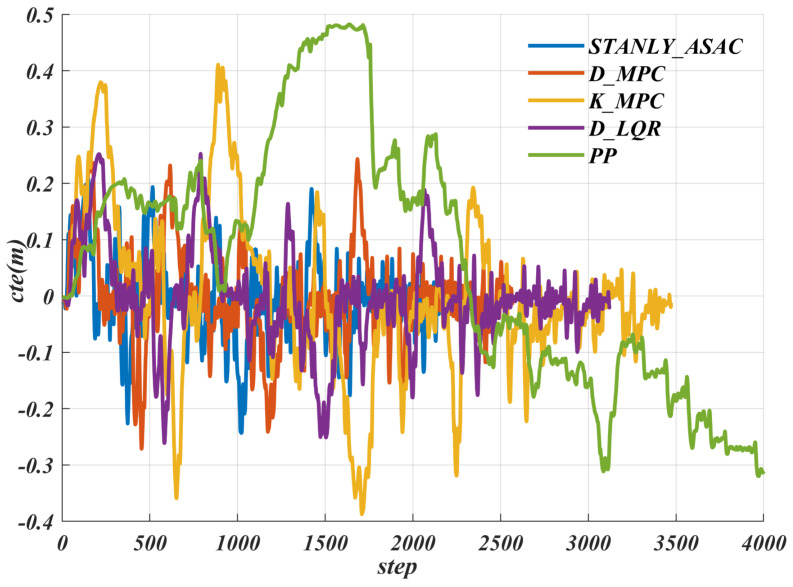
Comparative analysis of lateral error (cte) for various path−tracking algorithms on slippery water_ puddle road.

We conducted comparative path tracking experiments using the above different control algorithms on Water_Puddle roads. Based on Figure 16 and Table 7, the results showed that the proposed method can keep the cte within 0.25 even on slippery surfaces. In contrast, the maximum cte of the KMPC algorithm exceeds 0.4, and that of the PP algorithm is close to 0.5, which could significantly affect the safety of the robot during operation.

### 6.6. Energy Consumption

**Figure 17:**
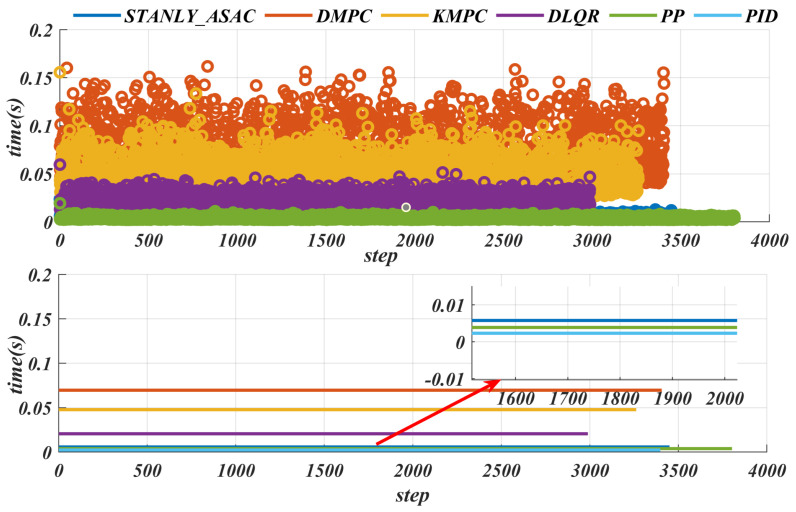
Actual path−tracking trajectories of different control algorithms on Water_Puddle road.

Efficient energy management can significantly improve the endurance, reliability, and energy efficiency of mobile robots operating in uncertain environments such as search and rescue [24,25]. Figure 17 shows the time consumption of different tracking algorithms under the same path and speed. The DMPC algorithm takes the longest time, with a maximum value exceeding 0.15 s (reaching 0.1642 s and an average time consumption of 0.0697 s). This is because DMPC is a nonlinear continuous model that requires linearization and discretization, and involves multivariable optimization, which results in a longer computation time. The KMPC algorithm uses a relatively simple model, but its variable parameters still need to be optimized and solved, so its time consumption is also high, with the maximum time also exceeding 0.15 s. In contrast, the PID control algorithm has the lowest time consumption. Since it does not require vehicle modeling, its maximum time consumption does not exceed 0.005 s. The maximum time consumption of the algorithm proposed in this paper is 0.0236 s, with an average time consumption of about 0.0978 s, and the control frequency exceeds 100 Hz. In addition, details of the cte are given in Table 8. The experimental results show that the controller consists of only a small number of modules, each with low computational complexity, resulting in negligible inter-module communication delays. Furthermore, since the robot operates at a low speed of 10 km/h, any delays between modules are minimal and have an insignificant impact on the overall control performance. Therefore, the effect of system delay on control performance can be neglected in this study.

## 7. Conclusions

In this paper, we propose a U-Net-based road semantic segmentation reinforcement learning controller to achieve accurate path tracking for autonomous vehicles on various road surfaces. First, we use a Lidar-IMU odometry framework to model the surrounding environment in 3D and output the vehicle’s positioning information in real time. Then, road surface detection information is obtained through U-Net semantic segmentation. Based on this information, we leverage the powerful learning capability of reinforcement learning to enable the controller to adapt to different road conditions. To reduce computational complexity, we introduce Stanley, a simple tracking controller based on a geometric algorithm, and combine it with an adaptive lateral deviation gain generated by reinforcement learning. This approach significantly reduces the computational burden and improves tracking efficiency while ensuring tracking accuracy. The experimental results show that our algorithm exhibits strong robustness against disturbances caused by changes in the road environment. This system achieves rapid response and correction of path-tracking errors through high-frequency IMU positioning and efficient controller design. The IMU’s output frequency of 100 Hz, combined with the controller’s average runtime of less than 0.0978 s, ensures that the control cycle is shorter than the positioning update interval. As a result, the system can correct trajectory deviations within 0.01 s, effectively improving path-tracking accuracy. In addition, we have conducted real-world experiments, and the results demonstrate that the algorithm performs well in terms of lightweight design, tracking accuracy, and real-time performance.

Limitations:

1. This method is limited by the tracking accuracy of the controller in extremely narrow passage scenarios [26]. If the passage width is less than the minimum distance required for the robot to pass safely, path planning becomes infeasible, thus limiting the applicability of the method in such environments [27].
2. This study has a real-time status-monitoring module that continuously monitors the output frequency of each subsystem and the lateral deviation during path tracking. If any indicator exceeds the preset threshold or the information has not been updated for a long time, the system will identify it as an abnormal situation, immediately stop the robot movement, and record the relevant data for subsequent fault analysis and system improvement. However, this monitoring mechanism may still have blind spots in extreme or unforeseen environments. Therefore, the safety and robustness of the system are limited by the coverage and sensitivity of the monitoring method, which is also a major limitation of this study.
3. This algorithm does not currently take road slope information into account, which to some extent limits its adaptability in complex road environments. Future work could incorporate slope information into the algorithm’s design to further enhance its adaptability to diverse road conditions.

Future work:

1. We plan to incorporate additional environmental information, such as slope and traffic conditions, to address more complex application scenarios. We will then conduct a more in-depth analysis of the impact of hyperparameters on controller performance to further enhance the algorithm’s generalization and adaptability.
2. We plan to increase the robot’s operating speed and introduce a trajectory prediction module for dynamic obstacles, thereby further enhancing the system’s adaptability and safety in complex environments.

## Figures and Tables

**Table 1:** Hardware specifications of the experimental platform.

Component	Manufacturer/Parameter
Robot	AgileX hunter se
Size	820 mm × 640 mm × 310 mm
Weight	42 kg
Battery	24 V 30 AH lithium
Motor	350 W*2 Brushless DC motor
Minimum turning radius	1.9 m
Turing method	Ackerman
Maximum speed	3 m/s
Lidar	RoboSense Helios-32 LiDAR
Camera	Hikvision MV-CA013-21UC
Lens	MVL-HF0628M-6MPE
IMU	INS-D-100
CPU	11^*th*^ Gen Intel Core i7-11700.2.5 GHZ
GPU	4070Ti, 12 GB

**Table 2:** Configuration of the U-Net.

Operational Layer (Type)	Output Shape
input_layer (InputLayer)	256, 256, 3
conv2d (Conv2D)	256, 256, 16
dropout (Dropout)	256, 256, 16
conv2d_1 (Conv2D)	256, 256, 16
max_pooling2d (MaxPooling2D)	256, 256, 16
conv2_2 (Conv2D)	128, 128, 32
dropout_1 (Dropout)	128, 128, 32
conv2_3 (Conv2D)	128, 128, 32
max_pooling2d_1 (MaxPooling2D)	64, 64, 32
conv2_4 (Conv2D)	64, 64, 64
dropout_2 (Dropout)	64, 64, 64
conv2_5 (Conv2D)	64, 64, 64
max_pooling2d_2 (MaxPooling2D)	32, 32, 64
conv2_6 (Conv2D)	32, 32, 128
dropout_3 (Dropout)	32, 32, 128
conv2_7 (Conv2D)	32, 32, 128
max_pooling2d_3 (MaxPooling2D)	16, 16, 128
conv2_8 (Conv2D)	16, 16, 256
dropout_4 (Dropout)	16, 16, 256
conv2_9 (Conv2D)	16, 16, 256
conv2_transpose (Conv2DTranspose)	32, 32, 128
concatenate (Concatenate)	32, 32, 256
conv2_10 (Conv2D)	32, 32, 128
dropout_5 (Dropout)	32, 32, 128
conv2_11 (Conv2D)	32, 32, 128
conv2_transpose_1 (Conv2DTranspose)	64, 64, 64
concatenate_1 (Concatenate)	64, 64, 128
conv2_12 (Conv2D)	64, 64, 64
dropout_6 (Dropout)	64, 64, 64
conv2_13 (Conv2D)	64, 64, 64
conv2_transpose_2 (Conv2DTranspose)	128, 128, 32
concatenate_2 (Concatenate)	128, 128, 64
conv2_14 (Conv2D)	128, 128, 32
dropout_7 (Dropout)	128, 128, 32
conv2_15 (Conv2D)	128, 128, 32
conv2_transpose_3 (Conv2DTranspose)	256, 256, 16
concatenate_3 (Concatenate)	256, 256, 32
conv2_16 (Conv2D)	256, 256, 16
dropout_8 (Dropout)	256, 256, 16
conv2_17 (Conv2D)	256, 256, 16
conv2_18 (Conv2D)	256, 256, 13

**Table 3:** Training parameters of the U-Net.

Hyperparameter	Value
Learning rate	0.0001
Optimizer	Adam
Batch size	8
Epochs	300
Lost function	Cross-Entropy Loss
Dropout	0.35
Video memory	12 GB
IoU (Intersection over Union)	93% for training set and 86% for validation set
Precision	0.95
Recall	0.93
Accuracy	0.95

**Table 4:** ASAC hyperparameter settings.

Hyperparameter	Value
Learning rate: actor	0.0003
Learning rate: critic	0.0003
Learning rate: entropy regularization coefficient	0.0003
Discount factor	0.99
Target network update rate	0.005
Replay buffer	1,000,000
Entropy regularization coefficient	0.2

**Table 5:** Comparison value of cte of path tracking under these four kinds of road.

Road Type	Max *cte*/m	Mean *cte*/m
Road_Asphalt	0.0919	0.0203
Paved_Road	0.0981	0.0217
Unpaved_Road	0.1057	0.0244
Water_Puddle	0.2054	0.0516

**Table 6:** Controller parameter setting table for each path tracking comparison algorithm.

Control Methods	Parameters
DMPC	Q[10 0 0; 0 100 0; 0 0 100] R[100],N_p(20),N_c(15)
KMPC	Q[10 0 0; 0 100 0; 0 0 100] R[100],N_p(15),N_c(10)
DLQR	Q[10 0 0; 0 100 0; 0 0 100] R[100]
PP	l_d(1.5)
PID	k_p(0.5),k_i(0.01),k_d(0.15)

**Table 7:** Comparison of cte of various path tracking algorithms under flooded road conditions.

Control Methods	Mean *cte*/m	Max *cte*/m
STANLY_ASAC	0.1036	0.2433
DMPC	0.0945	0.2713
KMPC	0.1416	0.4108
DLQR	0.1168	0.2612
pp	0.2318	0.4823

**Table 8:** Lateral error statistics of various path-tracking algorithms in energy management scenarios.

	*cte*/m		eψ/rad		*Time*/s	
** Controller**	**Max**	**Mean**	**Max**	**Mean**	**Max**	**Mean**
STANLY_SAC	0.2352	0.0668	0.1425	0.0644	0.0236	0.00978
DMPC	0.2403	0.0771	0.2063	0.1071	0.1642	0.0697
KMPC	0.4732	0.1341	0.3272	0.1456	0.1557	0.0478
DLQR	0.4859	0.1554	0.3660	0.1352	0.0596	0.0206
PP	0.4952	0.2385	0.3625	0.1245	0.0194	0.0039
PID	1.1264	0.3536	0.3895	0.1311	0.00429	0.0023

## Data Availability

The data that support the findings of this study are available from the corresponding author upon reasonable request.
